# Trehalose Alleviates Crystalline Silica-Induced Pulmonary Fibrosis via Activation of the TFEB-Mediated Autophagy-Lysosomal System in Alveolar Macrophages

**DOI:** 10.3390/cells9010122

**Published:** 2020-01-04

**Authors:** Xiu He, Shi Chen, Chao Li, Jiaqi Ban, Yungeng Wei, Yangyang He, Fangwei Liu, Ying Chen, Jie Chen

**Affiliations:** 1 Division of Pneumoconiosis, School of Public Health, China Medical University, No. 77 Puhe Road, Shenyang North New Area, Shenyang 110122, China; xiuhe_cmu@163.com (X.H.); lichao@cmu.edu.cn (C.L.); 17614027841@163.com (J.B.); weiyungeng1736@163.com (Y.W.); heyang129@sina.com (Y.H.); fwliu@cmu.edu.cn (F.L.); ychen25@cmu.edu.cn (Y.C.); 2 School of Medicine, Hunan Normal University, No.36 Lushan Road, Changsha 410013, China; chenshonest@163.com

**Keywords:** TFEB, trehalose, silicosis, alveolar macrophages, autophagy, lysosome

## Abstract

Silicosis is an occupational lung disease characterized by persistent inflammation and irreversible fibrosis. Crystalline silica (CS) particles are mainly phagocytized by alveolar macrophages (AMs), which trigger apoptosis, inflammation, and pulmonary fibrosis. Previously, we found that autophagy-lysosomal system dysfunction in AMs was involved in CS-induced inflammation and fibrosis. Induction of autophagy and lysosomal biogenesis by transcription factor EB (TFEB) nuclear translocation can rescue fibrotic diseases. However, the role of TFEB in silicosis is unknown. In this study, we found that CS induced TFEB nuclear localization and increased TFEB expression in macrophages both in vivo and in vitro. However, TFEB overexpression or treatment with the TFEB activator trehalose (Tre) alleviated lysosomal dysfunction and enhanced autophagic flux. It also reduced apoptosis, inflammatory cytokine levels, and fibrosis. Both pharmacologically inhibition of autophagy and TFEB knockdown in macrophages significantly abolished the antiapoptotic and anti-inflammatory effects elicited by either TFEB overexpression or Tre treatment. In conclusion, these results uncover a protective role of TFEB-mediated autophagy in silicosis. Our study suggests that restoration of autophagy-lysosomal function by Tre-induced TFEB activation may be a novel strategy for the treatment of silicosis.

## 1. Introduction

Silicosis is caused by the inhalation of respirable crystalline silica (CS), which causes persistent inflammation and irreversible fibrosis. Silicosis is a progressive condition that is almost always fatal even when exposure to CS dust has ceased [1,2,3]. It is generally accepted that alveolar macrophages (AMs) are the first and main target cells to contact with CS particles [4]. The primary function of resident AMs is to keep the air spaces clear by the removal of foreign substances; however, CS cannot be degraded and eventually causes fibrosis [5,6]. Apoptosis of AMs appears to play a critical role in silicosis. Apoptotic AMs release their contents, which can aggravate apoptosis and the inflammatory response to induce tissue damage and fibrosis [7]. Moreover, apoptotic AMs release ingested CS particles that are re-phagocytosed by healthy AMs, resulting in a cycle of persistent inflammation, and lung tissue damage [8,9]. 

Autophagy is an evolutionarily conserved cellular process that maintains cell homeostasis by disposing of denatured proteins and damaged organelles through a lysosomal degradation pathway [10,11]. Lysosomes fuse with phagosomes to degrade their contents, which contributes to lysosomal membrane permeabilization (LMP) [4,12]. Increased lysosome injury is observed in AMs in response to the uptake of CS particles. Previously, we showed that CS-induced lysosome destruction impaired autophagic substrate degradation in the AMs of silicosis patients [13]. However, the mechanism by which AM autophagy plays a role in silicosis is still unclear. An increased number of autophagosomes has been observed in the CS-induced damage to macrophages [13,14,15,16]. Therefore, restoring the autophagy-lysosomal system function and enhancing the autophagic flux could provide a molecular target to delay CS-induced persistent inflammation and fibrosis. 

Transcription factor EB (TFEB) is a master regulator of autophagy-related gene transcription and lysosome biogenesis [17,18,19]. TFEB activation is efficacious against inflammatory and fibrotic diseases [20,21]. Trehalose (Tre) is an mTOR-independent inducer of autophagy that can improve the clinical symptoms of neurodegenerative disorders in animal models [22]. In atherosclerosis, Tre induced macrophage autophagy-lysosomal biogenesis and produced similar effects to TFEB overexpression in macrophages [23,24]. However, the roles of TFEB and Tre in CS-induced pulmonary inflammation and fibrosis have not been explored. Therefore, in this study, we assessed the role of TFEB in silicosis models and investigated whether Tre could alleviate CS-induced inflammation and fibrosis via the activation of the TFEB-mediated autophagy-lysosomal system.

## 2. Materials and Methods

### 2.1. Antibodies and Other Reagents

The following antibodies were used for immunofluorescence (IF), immunoblotting (IB), or immunohistochemistry (IHC) in preset study: β-Actin (4967, Cell Signaling Technology [CST], Danvers, MA, USA, 1:1000 for IB); lamin B (12987-1-AP, Proteintech, Philadelphia, PA, USA, 1:1000 for IB); F4/80 (sc-377009, Santa Cruz Biotechnology, Santa Cruz, CA, USA, 1:50 for IF); TFEB (A303-673A-M, Bethyl Laboratories, Montgomery, AL, USA, 1:1000 for IB and 133721-1-AP, Proteintech, Philadelphia, PA, USA, 1:100 for IF, 1:200 for IHC); LC3 (12741, CST, Danvers, MA, USA, 1:1000 for IB and PM036, Medical & Biological Laboratories [MBL], Aichi, Japan, 1:200 for IF); Atg5 (129994, CST, Danvers, MA, USA, 1:1000 for IB); ubiquitin (10201-2-AP, Proteintech, Philadelphia, PA, USA, 1:500 for IB and sc-8017, Santa, Santa Cruz, CA, USA;1:50 for IF); caspase-3 (Cas3) (19677-1-AP, Proteintech, Philadelphia, PA, USA, 1:1000 for IB); BAX (50599-1-lg, Proteintech, Philadelphia, PA, USA, 1:500 for IB), BCL-2 (12789-1-AP, Proteintech, Philadelphia, PA, USA, 1:1000 for IB); cathepsin B (CTSB) (31718, CST, Danvers, MA, USA, 1:1000 for IB); LAMP1 (ab24170, abcam, Cambridge, UK, 1:1000 for IB and 1:200 for IF); collagen I (Col-1) (WL0088, Wanleibio, Liaoning, China, 1:500 for IB and abs131984, Absin Bioscience, Shanghai, China, 1:100 for IHC); fibronectin (Fn) (WL03180, Wanleibio, Liaoning, China, 1:500 for IB and NBP1-91258ss, Novus biological, Littleton, CO, USA, 1:200 for IHC); Beclin 1 (3738S, CST, Danvers, MA, USA, 1:1000 for IB); p62 (ab109012, abcam, Cambridge, UK, 1:10000 for IB); peroxidase-conjugated goat anti-rabbit IgG (H+L) (ZB2301, ZSGB-BIO, Beijing, China, 1:5000 for IB); rhodamine (TRITC)-conjugated goat anti-rabbit IgG (H+L) (ZB0316, ZSGB-BIO, Beijing, China, 1:200 for IF); fluorescein-conjugated goat anti-rabbit IgG (H+L)(ZF0311, ZSGB-BIO, Beijing, China, 1:200 for IF); rhodamine (TRITC)-conjugated goat anti-mouse IgG (H+L) (ZF-0313, ZSGB-BIO, Beijing, China, 1:200 for IF); and Alexa Fluor^®^ 488 AffiniPure Fab Fragment Goat Anti-rabbit IgG (H+L) (111-547-003, Jackson ImmunoResearch, West Grove, PA, USA, 1:100 for IF). 

The following reagents were used in this study: LysoTracker Red (C1046, Beyotime, Shanghai, China, 50 nmol/L); acridine orange (A6014, Sigma, Saint Louis, MO, USA, 5 μg/mL); One-Step TUNEL Apoptosis Assay Kit (C1088, Beyotime, Shanghai, China); 3-methyladenine (3MA) (HY-19312, MedChemExpress [MCE], Monmouth Junction, NJ, USA, 0.5 mmol/L); bafilomycin A1 (BAF) (HY-100558, MCE, Monmouth Junction, NJ, USA, 10 nmol/L); Cell Counting Kit-8 (HY-K0301, MCE, Monmouth Junction, NJ., USA); chloroquine (CQ) (C6628, Sigma, Saint Louis, MO, USA, 2 µmol/L); and trehalose (90210, Sigma, Saint Louis, MO, USA, 2 mg/g for animals and T0167, Sigma, Saint Louis, MO, USA, 50 mmol/L for MH-S cells). 

### 2.2. Animals and Treatment

Male C57BL/6 mice (18 to 22 g, 6 to 8 weeks) were purchased from SLAC Laboratory Animal Co. Ltd. (Shanghai, China) with National Animal Use License number SYXK-LN2013-0007. All animal protocols were approved by the Animal Care and Use Committee of the China Medical University and conducted in compliance with the National Institutes of Health Guide for Care and Use of Laboratory Animals. All efforts were made to minimize the number of animals used and their suffering. In this study, the mice were randomly divided into the following four treatment groups: (1) Control (Ctrl) group, mice received saline intraperitoneally (i.p.) after intratracheal injection of saline; (2) trehalose (Tre) group, mice received 2 g/kg trehalose in saline i.p. after intratracheal injection of saline; (3) crystalline silica (CS) group, mice received saline i.p. after intratracheal injection of crystalline CS suspended in saline; and (4) crystalline silica plus trehalose (CS + Tre) group, mice received 2 g/kg trehalose i.p. after intratracheal injection of CS suspended in saline. CS particulates (Min-U-Sil 5 ground silica with size distribution of 97% < 5 μm diameter, 80% < 3 μm diameter, and median diameter 1.4 µm) were purchased form the U.S. Silica Company (Frederick, MD, USA). The pretreatment of CS and the establishment of silicosis animal models were performed as previously described [25,26]. The schematic representation for the in vivo treatment schedule is presented in Figure S1. Mouse lungs were collected 7 or 56 days after CS and Tre administration.

### 2.3. Isolation of Primary Alveolar Macrophages (AMs)

AMs were collected from bronchoalveolar lavage fluid (BALF) as described previously [15]. Briefly, the BALF was centrifuged at 4 °C and 1000× *g* for 10 min. The supernatant was transferred to a new centrifuge tube for the detection of protein concentration and cytokines. Cells were resuspended in RPMI-medium containing 10% fetal bovine serum (heat-inactivated), 2 mmol/L L-glutamine (Invitrogen, Waltham, MA, USA), and 1000 U/mL penicillin-streptomycin and incubated for 2 h at 37 °C and 5% CO_2_. After the incubation, the non-attached cells were discarded, and the adherent AMs were collected and used for analysis. 

### 2.4. Cell Culture and Treatment

The mouse AM cell line MH-S (National Infrastructure of Cell Line Resource, Beijing, China) was cultured with RPMI medium supplemented with 10% heat-inactivated fetal calf serum, penicillin (100 U/mL), and streptomycin (100 µg/mL) in a 37 °C incubator and 5% CO_2_. For the in vitro experiments, MH-S cells were treated for 12 h with medium containing 50 μg/cm^2^ CS, 50 mmol/L Tre, or both agents.

### 2.5. Lentiviral Short Hairpin RNA (shRNA) Transfection

The TFEB expression vector and TFEB or negative control lentiviral shRNA constructs were obtained from Hanbio Company (Shanghai, China). MH-S cells were transfected with lentiviral shRNA to knockdown or overexpress TFEB (multiplicity of infection, MOI = 30) according to the manufacturer’s protocols. MH-S cells were transfected with the TFEB lentiviral shRNA and negative control in the same process and dose. The shRNA sequences were as follows: TFEB, 5′- CCAAGAAGGATCTGGACTT-3′ and negative control, 5′-TTCTCCGAACGTGTCACGTAA-3′. The transfection efficiency was determined by immunoblotting (Figure S2). 

### 2.6. Cell Viability Assay

MH-S Cell viability was determined using Cell Counting Kit-8 (CCK8). Briefly, 5 × 10^5^ cells/mL were plated in 96-well plates. The cells were incubated with different concentrations of the autophagy inhibitors BAF, CQ, or 3MA (Figure S3) or Tre (Figure S4). CCK8 solution (10 μL) was added to the treated cells in each well, and the cells were incubated for 1 to 4 h at 37 °C and 5% CO_2_. The absorbance of each well was determined using a microplate reader (Tecan, Shanghai, China). 

### 2.7. mRFP-GFP-LC3 Transfection

MH-S cells were transfected with adenovirus plasmid mRFP-GFP-LC3 (Hanbio Biotech, Shanghai, China), according to the manufacturer’s protocol. After the indicated treatments, cells were fixed with 4% paraformaldehyde for 5 min, and images captured using laser scanning confocal microscopy (A1R, Nikon, Tokyo, Japan). Because of the acid dissolution property of GFP, the transformation of the autophagosomes to autolysosomes can be observed as the conversion of yellow and red fluorescence in real-time [27]. 

### 2.8. Lysotracker Red and Acridine Orange Staining

Lysotracker Red and acridine orange staining were used to assess the functional state of lysosomes. The MH-S cells were seeded on coverslips in 24-well plates. After treatment, the cells were incubated in medium containing 50 nmol/L Lysotracker Red or 5 μg/mL acridine orange hydrochloride for 30 min at 37 °C and 5% CO_2_ [28]. The coverslips were then washed three times and observed using fluorescence microscopy (Nikon, Tokyo, Japan). 

### 2.9. Enzyme-Linked Immunosorbent Assay (ELISA)

The levels of cytokines IL-6, MCP-1, TNF-α, and IL-1β in the MH-S cell supernatant and BALF were quantified by ELISA (R&D Systems, Minneapolis, MN, USA), according to the manufacturer’s instructions. For the evaluation of IL-1β levels, the cells were pretreated with LPS (25 ng/mL, Sigma, Saint Louis, MO, USA) for 3 h before treatment [29]. The absorbances at 450 nm and 570 nm were measured using a microplate reader (Tecan, Shanghai, China).

### 2.10. Immunoblotting

Nuclear and cytoplasmic proteins from mouse lung tissue or treated MH-S cells were extracted using the Nuclear and Cytoplasmic Protein Extraction Kit (P0028, Beyotime, Shanghai, China), according to the manufacturer’s instructions. Protein concentration was measured using the BCA kit (Beyotime, Shanghai, China). An equal volume of methanol and 0.25 volumes of chloroform were used to extract protein from the BALF or cell culture supernatants. Samples were vortexed and centrifuged for 10 min at 20,000× *g*. The upper phase was removed, and methanol (500 μL) was added to the interphase. The mixture was centrifuged for 10 min at 20,000× *g*, and the protein pellet was dried, resuspended in loading buffer, and boiled for 5 min at 100 °C [6,30]. The samples were diluted to a concentration of 3 μg/μL. The protein samples (30 μg) were separated on SDS-PAGE gels and then transferred onto PVDF membranes (Millipore, Billerica, MA, Germany). After blocking with 5% defatted dried milk in TBST, the membranes were incubated overnight at 4 °C with primary antibodies against TFEB, BAX, BCL-2, Beclin 1, Atg5, p62, LC3, LAMP1, caspase-3, CTSB, Col-1, Fn, β-actin, or lamin B. Subsequently, the membranes were incubated with horseradish peroxidase-conjugated secondary antibody and rinsed with TBST. The specific proteins were observed using the version C500 Bioanalytical Imaging System (Azure Biosystems, Inc., Dublin, CA, USA). Image J software was used to quantify the density of the protein bands. 

### 2.11. Immunofluorescence

The lung sections were washed using phosphate-buffered saline after deparaffinization and rehydration. The treated MH-S cells were fixed in 4% formaldehyde for 30 min at room temperature and permeabilized with 0.2% Triton X-100 for 20 min. The sections and cells were blocked with 5% bovine serum albumin at room temperature. The samples were incubated with primary antibodies against TFEB, LC3, p62, LAMP1, ubiquitin, or F4/80 overnight at 4 °C followed by incubation with secondary antibodies for 2 h at room temperature in the dark. After washing, the cells were mounted with ProLong^®^ Diamond Antifade Mountant, containing DAPI (ThermoFisher, Waltham, MA, USA). Images were collected by fluorescence microscopy (Nikon, Tokyo, Japan).

### 2.12. Quantitative PCR (qPCR)

Total RNA was extracted from lung tissue or treated cells using Trizol (Takara, Tokyo, Japan), according to the manufacturer’s protocol. Reverse transcription was performed using the Prime Script RT kit (Takara, Japan), and qPCR was conducted using the SYBR Green Master Mix Kit (Takara, Japan). The data were normalized to GAPDH as the endogenous control. Relative expression was calculated using the 2^−ΔΔCt^ method. The specific primer sequences were as follows: TFEB, 5′-CACAGGTTACCCCGATACC-3′ and 5′-AGGGAGTCATCTAGGAGCATTA-3′; GAPDH, 5′-CAATGTGTCCGTCGTGGATCT-3′ and 5′-GTCCTCAGTGTAGCCCAAGATG-3′.

### 2.13. Histology and Immunohistochemistry

Inflammation and fibrosis were assessed by hematoxylin and eosin (HE) or Masson’s trichrome staining of paraffin sections (5 μm) of lung tissue, according to the manufacturer’s protocol. For immunochemistry, the lung sections were incubated with primary antibodies against Col-1 and Fn overnight at 4 °C. After washing, the sections were stained with HRP-conjugated secondary antibodies, followed by visualization with diaminobenzidine. Fibrosis was scored using Image-Pro Plus version 6.0 software (Media Cybernetics, Rockville, MD, USA). Three different fields within a lung section and three sections per animal (*n* = 4 to 5 mice/group) were evaluated to obtain a mean value.

### 2.14. TUNEL Assay

The percentage of apoptotic MH-S cells following treatment was determined using terminal deoxynucleotidyl transferase-mediated dUTP nick end labeling staining (TUNEL), according to the manufacturer’s instructions. The cells were fixed with 4% paraformaldehyde and permeabilized with 0.2% Triton X-100. Images were collected using fluorescence microscopy (Nikon, Japan).

### 2.15. Statistical Analysis

All numerical data are presented as the mean ± standard deviation (SD). Statistical significance was determined using the unpaired two-sided Student’s *t*-test (for two groups) or one-way analysis of variance (ANOVA) followed by the Student–Newman–Keuls test (for multiple groups). A value of *p* < 0.05 indicated statistical significance. The statistical analyses were conducted using SPSS version 19.0 software (SPSS Inc., Chicago, IL, USA).

## 3. Results

### 3.1. CS Induces TFEB Nuclear Localization and Increases TFEB Expression In Vivo

**Figure 1:**
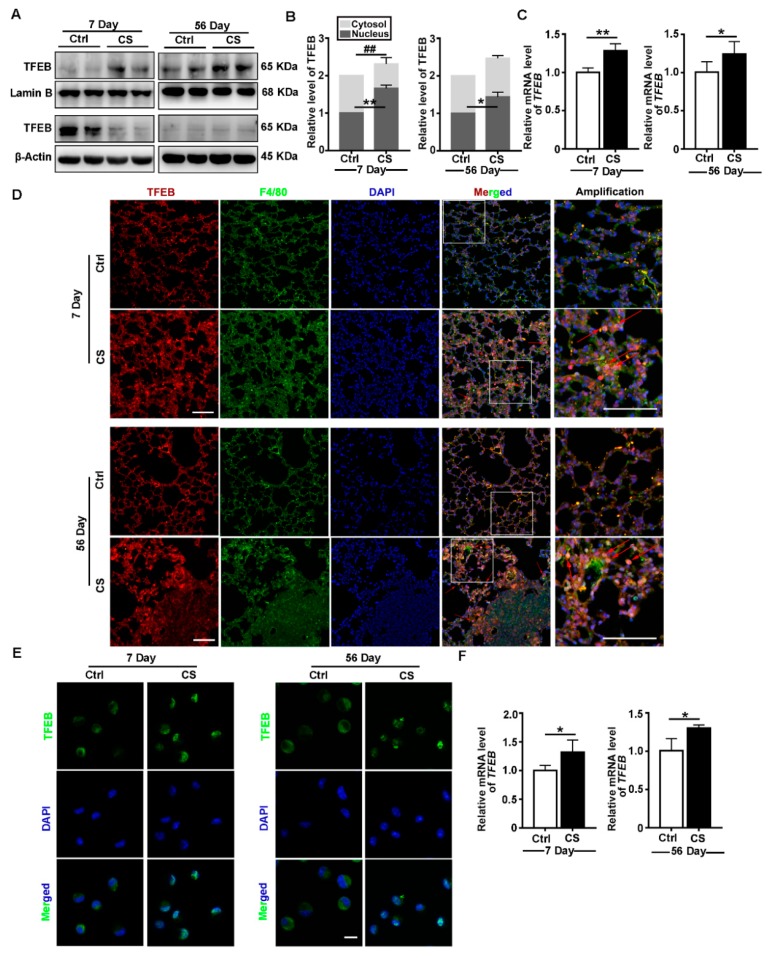
Crystalline silica (CS) induces transcription factor EB (TFEB) nuclear localization and increases TFEB expression in vivo. (**A**,**B**) Immunoblotting analysis of TFEB levels in the cytosol or nucleus of lung tissue on day 7 or day 56 post CS instillation; β-actin, loading control for cytosolic proteins; and lamin B, loading control for nuclear proteins (*n* = 4). (**C**) qPCR analysis of TFEB expression in lung tissue on day 7 or day 56 (*n* = 5 to 6). (**D**) Immunofluorescence analysis of the distribution of F4/80 and TFEB in lung tissue on day 7 or 56 post saline or CS treatment. Red arrows indicate alveolar macrophages (AMs) in the lungs containing silicotic lesions with predominantly nuclear TFEB staining (scale bar = 50 μm and *n* = 5). (**E**) Immunofluorescence analysis of TFEB nucleus translocation in primary AMs from bronchoalveolar lavage fluid (BALF) (scale bar = 10 μm and *n* = 3 to 4). (**F**) qPCR analysis of TFEB expression in primary AMs on day 7 or 56 post CS instillation (*n* = 4). Data are presented as the mean ± SD. *, *p* < 0.05; **, *p* < 0.01; and ##, *p* < 0.01.

TFEB belongs to the MiT-TFE family of basic helix–loop–helix leucine-zipper transcription factors, which plays a vital role in the adaptation of cell homeostasis [31]. To explore the role of TFEB in inflammation and fibrosis, we examined TFEB expression in the lungs of CS-treated or control mice on day seven (inflammation stage) or day 56 (fibrosis stage) post CS treatment. As shown in Figure 1A–D, CS caused the nuclear translocation of TFEB and upregulation of TFEB expression in lung tissues. In particular, TFEB nuclear localization was more evident in alveolar macrophages (AMs) present in silicotic lesions as compared with the control tissue (Figure 1D). Then, we confirmed nuclear localization and upregulated expression of TFEB in AMs of model mice by immunofluorescence and qPCR (Figure 1E,F). Thus, the nuclear translocation and increased expression of TFEB in AMs could be involved in CS-induced inflammation and fibrosis.

### 3.2. TFEB Modulates CS-Induced Macrophages Apoptosis and Secretion of Inflammatory Cytokines In Vitro

**Figure 2:**
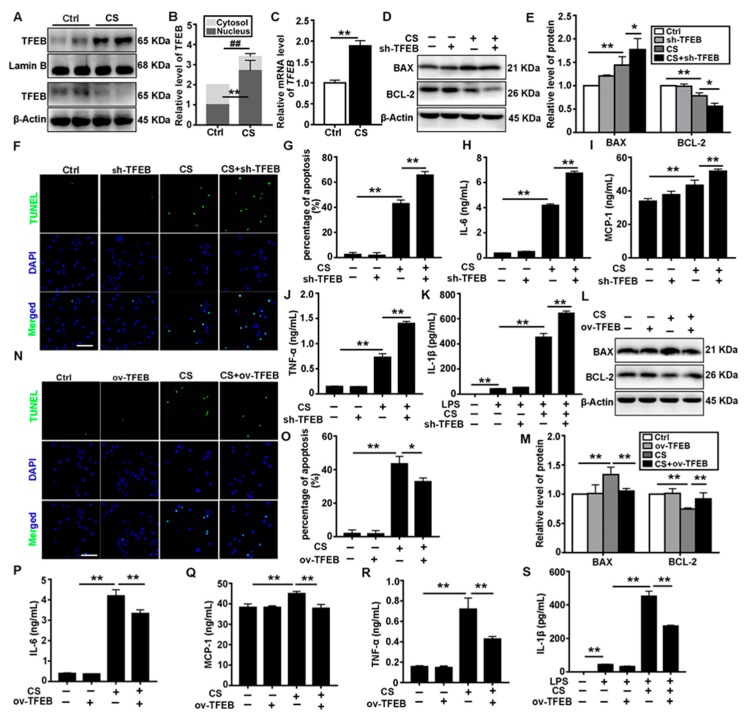
TFEB modulates CS-induced macrophage apoptosis and inflammatory response in vitro. (**A**,**B**) Immunoblotting analysis of TFEB levels in the cytosol and nucleus of MH-S cells 12 h post saline or CS treatment; β-actin, loading control for cytosolic proteins and lamin B, loading control for nuclear proteins (*n* = 4). (**C**) qPCR analysis of TFEB expression in MH-S cells 12 h post-saline or CS treatment (*n* = 4). (**D**,**E**) TFEB knockdown deteriorates CS-induced apoptosis and inflammation. Immunoblotting analysis of BAX and BCL-2 protein levels (*n* = 3 to 4). (**F**,**G**) TUNEL (green) and DAPI (blue) staining and ratios of TUNEL-positive apoptotic cells (scale bar = 50 μm and *n* = 4). (**H**–**K**) ELISA analysis of IL-6, MCP-1, TNF-α, and IL-1β levels in MH-S cell supernatant (*n* = 3 to 4). (**L**,**M**) TFEB overexpression relieves CS-induced apoptosis and inflammation. Immunoblotting analysis of BAX and BCL-2 protein levels (*n* = 4). (**N**,**O**) TUNEL (green) and DAPI (blue) staining and ratios of TUNEL-positive apoptotic cells (scale bar = 50 μm and *n* = 4). (**P**–**S**) ELISA analysis of IL-6, MCP-1, TNF-α, and IL-1β levels in MH-S cell supernatant (*n* = 3 to 4). Data are presented as the mean ± SD. *, *p* < 0.05; **, *p* < 0.01; and ##, *p* < 0.01.

Because nuclear localization of TFEB was observed in AMs in vivo after CS treatment, we explored the effects of CS exposure on TFEB in vitro. Consistent with the in vivo data, expression of TFEB was upregulated in response to CS, and TFEB protein was localized in the nucleus of MH-S cells (Figure 2A–C). To further elucidate the role of TFEB in CS-treated macrophages, we established MH-S cell lines with either TFEB knockdown or overexpression. The apoptosis of AMs plays a critical role in the etiology of silicosis. BAX and BCL-2 are two members of the BCL-2 protein family, which are pro-and antiapoptotic proteins, respectively [32]. We found that CS treatment reduced BCL-2 levels but augmented BAX levels, leading to an increase apoptosis in AMs. CS treatment also increased the secretion of inflammatory cytokines, including IL-6, MCP-1, TNF-α, and IL-1β. However, TFEB knockdown markedly exacerbated the apoptosis and inflammatory cytokines secretion induced by CS treatment (Figure 2D–K). In contrast, TFEB overexpression reversed the detrimental effects of CS (Figure 2L–S). 

### 3.3. TFEB Overexpression Abrogates CS-Induced Macrophage Apoptosis and Secretion of Inflammatory Cytokines via Regulation of the Autophagy-Lysosome Pathway

**Figure 3:**
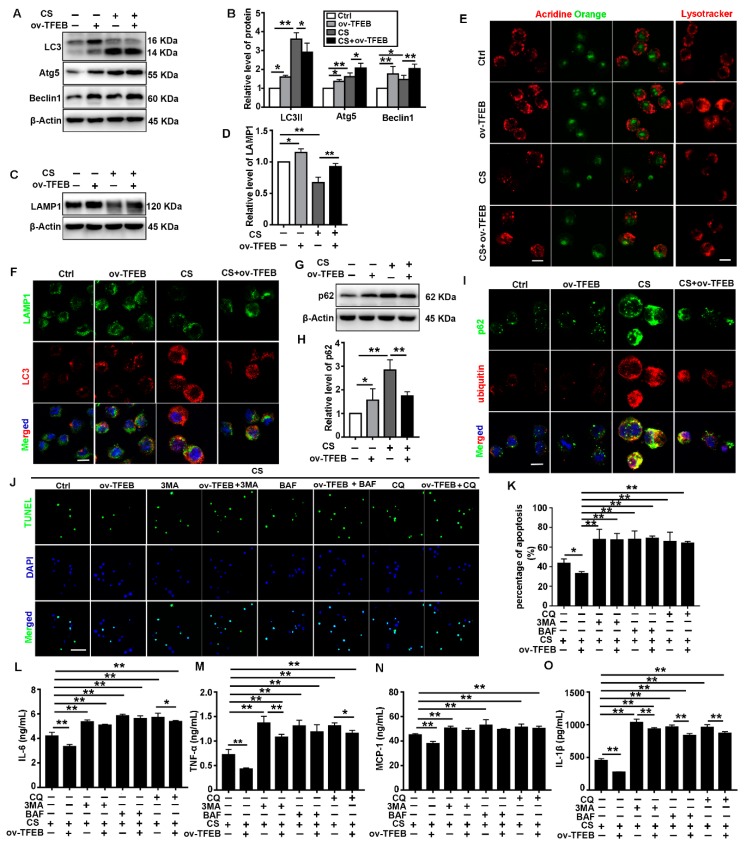
TFEB overexpression relieves CS-induced macrophage apoptosis and inflammation via autophagic substrate degradation in vitro. (**A**,**B**) Immunoblotting analysis of LC3II, Atg5, and Beclin 1 protein levels (*n* = 4). (**C**,**D**) Immunoblotting analysis of LAMP1 protein levels (*n* = 3). (**E**) Cells were grown on coverslips and treated with CS followed by staining with 50 nmol/L Lysotracker Red or 5 μg/mL acridine orange at 37 °C for 30 min (scale bar = 10 μm and *n* = 4). (**F**) Immunofluorescence analysis of the colocalization of LAMP1 and LC3 12 h post CS treatment (scale bar = 25 μm and *n* = 4). (**G**,**H**) Immunoblotting analysis of p62 protein levels (*n* = 4). (**I**) Immunofluorescence analysis of the colocalization of the p62 and ubiquitin 12 h post CS treatment (scale bar = 25 μm and *n* = 4). **(J**,**K**) TUNEL (green) and DAPI (blue) staining and ratios of TUNEL-positive apoptotic cells (scale bar = 50 μm and *n* = 4). (**L**–**O**) ELISA analysis of IL-6, MCP-1, TNF-α, and IL-1β levels in MH-S cell supernatant (*n* = 3 to 4). Data are presented as the mean ± SD. *, *p* < 0.05 and **, *p* < 0.01.

TFEB is a key regulator of the autophagy-lysosome system. Thus, we examined whether the autophagy-lysosome system was involved in the antiapoptotic and anti-inflammatory effect of TFEB overexpression. As shown in Figure 3A,B, TFEB overexpression stimulated CS-induced upregulation of Atg5 and Beclin 1 in MH-S cells. In contrast, it decreased the CS-induced upregulation of LC3II. These data suggest that TFEB overexpression could promote the production of autophagy-related proteins and facilitate the degradation of autophagic substrates in CS-treated MH-S cells. We also assessed the synthesis and degradation of autophagic substrates after CS treatment using BAF and CQ. We found that the CS-induced increase in the number of autophagosomes occurred due to both autophagy activation and inhibition of cargo degradation (Figure S5). 

Lysosomes are normally the degradative compartments for autophagic substrates. However, lysosomal dysfunction leads to the deposition of autophagosomes [33]. In this study, TFEB overexpression alleviated the CS-induced reduction in LAMP1 levels and red fluorescence (Figure 3C–E). Furthermore, TFEB overexpression restored the colocalization of LAMP1 and LC3 that was disrupted by CS (Figure 3F). These results suggest that TFEB overexpression attenuate the CS-induced inhibition of autophagosome-lysosome fusion. Lysosomal degradation of cargo is a crucial process in autophagic flux. TFEB overexpression decreased the accumulation of p62 and ubiquitinated proteins induced by CS treatment (Figure 3G–I). Thus, TFEB overexpression could mitigate the inhibition of autophagic substrate degradation caused by CS treatment. To further investigate the role of TFEB in this process, we determined the effect of autophagy inhibitors (BAF, CQ, and 3-MA) on the autophagic flux mediated by TFEB overexpression. The autophagy inhibitors abolished the protective effects of TFEB overexpression on CS-induced apoptosis and inflammatory cytokine release (Figure 3J–O). These results confirm that TFEB overexpression blocks CS-induced macrophage apoptosis and inflammatory cytokine secretion by regulating the autophagy-lysosome pathway.

### 3.4. Tre Activates TFEB and Affects Autophagy-Associated Proteins in a Silicosis Mouse Model

**Figure 4:**
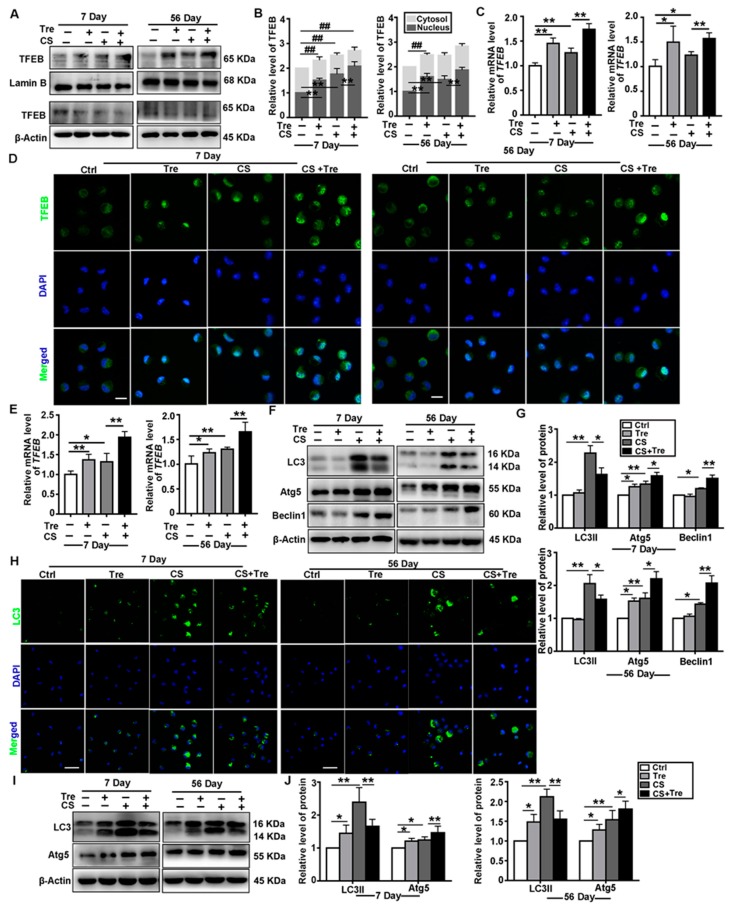
Trehalose (Tre) affects autophagy-associated proteins in the lungs and AMs of CS-treated mice. (**A**,**B**) Immunoblotting analysis of cytosol and nuclear TFEB levels in lung tissue on day 7 or 56 post CS or Tre treatment; β-actin, loading control for cytosolic proteins; and LaminB, loading control for nuclear proteins (*n* = 3 to 4). (**C**) qPCR analysis of TFEB levels in lung tissue on day 7 or 56 post-CS or Tre treatment (*n* = 4). (**D**) Immunofluorescence analysis of TFEB nuclear translocation in primary AMs in BALF on day 7 or 56 (scale bar = 10 μm and *n* = 3 to 4). (**E**) qPCR analysis of TFEB expression in AMs on day 7 or 56 post CS or Tre treatment (*n* = 4). (**F**,**G**) Immunoblotting analysis of LC3II, Atg5, and Beclin 1 protein levels in lung tissue on day 7 or 56 post CS and Tre treatment (*n* = 5 to 6). (**H**) Immunofluorescence analysis of LC3 in AMs on day 7 or 56 post CS or Tre treatment (Scale bar = 25 μm and *n* = 3). (**I**,**J**) Immunoblotting analysis of LC3II and Atg5 protein levels in AMs on day 7 or 56 post CS or Tre treatment (*n* = 3 to 4). Data are presented as the mean ± SD. *, *p* < 0.05; **, *p* < 0.01; and ##, *p* < 0.01.

Tre is an inducer of autophagy-lysosomal biogenesis by an unclear mechanism. It has demonstrated anti-inflammatory effects in many diseases [34,35,36]. In this study, Tre induced TFEB nuclear localization and upregulated TFEB expression in the lungs (Figure 4A–C) and AMs (Figure 4D–E) of CS-treated mice. CS exposure caused the upregulation of LC3II, Atg5, and Beclin 1 expression in the lungs of the mice. Tre also induced the upregulation of Atg5 and Beclin 1 levels but downregulated LC3II after CS exposure (Figure 4F,G). Similar results were observed with primary AMs (Figure 4H–J). These data suggest that Tre could promote the degradation of autophagic substrates in CS-treated cells through its effects on TFEB nuclear localization and expression.

### 3.5. Tre Relieves CS-Induced Lysosome Damage and Disorder of Autophagic Substrates Degradation In Vivo

**Figure 5:**
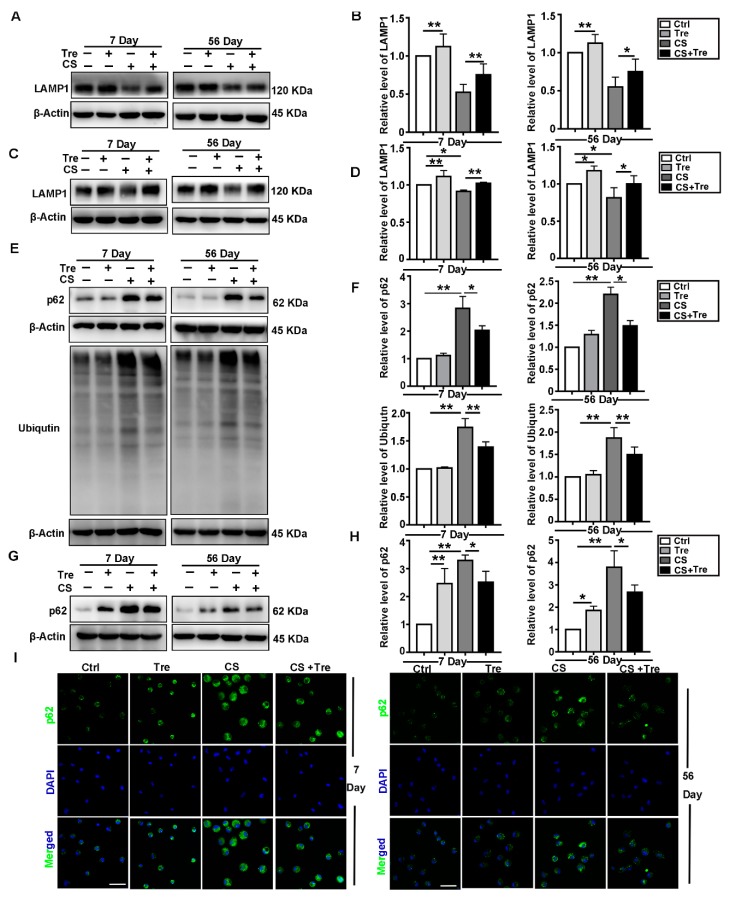
Tre relieves CS-induced lysosome damage and disorder of autophagic substrate degradation in vivo. (**A**,**B**) Immunoblotting analysis of LAMP1 protein levels in lung tissue on day 7 or 56 post CS instillation (*n* = 5 to 6). (**C**,**D**) Immunoblotting analysis of LAMP1 protein levels in AMs on day 7 or 56 post CS instillation (*n* = 3 to 4). (**E**,**F**) Immunoblotting analysis of p62 and ubiquitin protein levels in lung tissue on day 7 or 56 post CS instillation (*n* = 5 to 6). (**G**,**H**) Immunoblotting analysis of p62 protein levels in AMs on day 7 or 56 post CS instillation (*n* = 3 to 4). (**I**) Immunofluorescence analysis of p62 in AMs on day 7 or 56 post CS instillation (scale bar = 25 μm and *n* = 3). Data are presented as the mean ± SD. *, *p* < 0.05 and **, *p* < 0.01.

Tre modulates lysosomal function to accelerate the degradation of autophagic substrates [37,38]. In our study, we used LAMP1 and cathepsin B (CTSB) to monitor lysosomal function in the lungs of CS-treated mice, primary AMs, or BALF. We found that Tre reversed the decrease in LAMP1 levels caused by CS treatment in the lungs or primary AMs at 7 or 56 days post CS treatment (Figure 5A–D). Furthermore, Tre alleviated the CS-induced increases in the CTSB levels in the BALF (Figure S6A). CS also upregulated the expression of ubiquitinated proteins in lung tissue and p62 levels in both lung tissue and primary AMs. Tre attenuated the accumulation of these proteins (Figure 5E–I). These data suggest that Tre alleviates the CS-induced lysosomal damage and promotes the degradation of autophagic substrates.

### 3.6. Tre Activates TFEB and Relieves CS-Induced Lysosomes Damage and Disorder of Autophagic Substrate Degradation through TFEB Activation In Vitro

**Figure 6:**
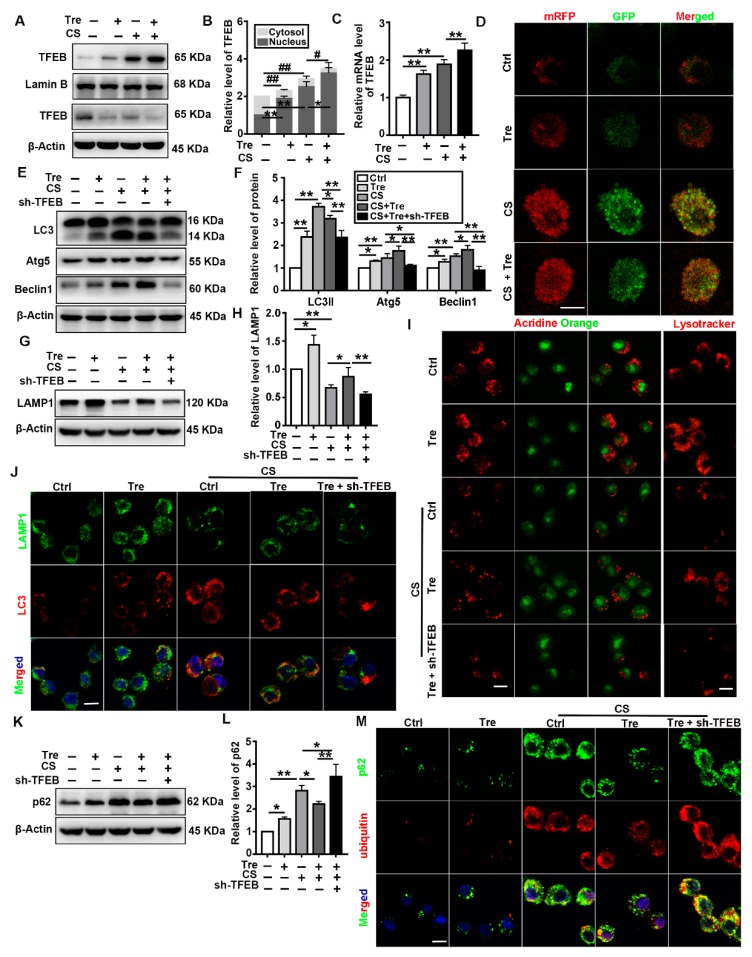
Tre relieves CS-induced lysosome damage and restores autophagic substrate degradation through TFEB activation in vitro. (**A**,**B**) Immunoblotting analysis of TFEB levels in the cytosol and nucleus of MH-S cells post CS and Tre treatment and β-actin, loading control for cytosolic proteins; LaminB, loading control for nuclear proteins (*n* = 3 to 4). (**C**) qPCR analysis of TFEB expression in MH-S cells 12 h post-CS and Tre treatment (*n* = 3 to 4). (**D**) MH-S cells were transfected with the adenovirus mRFP-GFP-LC3 plasmid. LC3 puncta were observed by confocal microscopy (scale bar = 10 μm and *n* = 3). (**E**,**F**) Immunoblotting analysis of LC3II, Atg5, and Beclin 1 protein levels (*n* = 3). (**G**,**H**) Immunoblotting analysis of LAMP1 protein levels in cell lysates (*n* = 4). (**I**) Cells were grown on coverslips and stained with Lysotracker Red or acridine orange 12 h post CS and Tre treatment (scale bar = 10 μm and *n* = 3). (**J**) Immunofluorescence analysis of the colocalization of LAMP1 and LC3 12 h post CS and Tre treatment (scale bar = 25 μ and *n* = 3). (**K**–**M**) Immunoblotting analysis of p62 and immunofluorescence analysis of the colocalization of p62 and ubiquitin 12 h post CS and Tre treatment (scale bar = 25 μm and *n* = 3). Data are presented as the mean ± SD. *, *p* < 0.05; **, *p* < 0.01; #, *p* < 0.05; and ##, *p* < 0.01.

Because Tre protected the autophagy-lysosome system in CS-treated mice, we explored the possible mechanisms underlying these protective effects in vitro. CS-induced TFEB nuclear localization and expression were significantly heightened by Tre treatment (Figure 6A–C). Subsequently, we used mRFP-GFP-LC3 adenovirus-infected MH-S cells to monitor autophagic flux. Because of the acid dissolution property of GFP, the transformation of the autophagosomes into autolysosomes can be visualized as a fluorescence conversion in real time. In the merged images of Figure 6D, the red and yellow dots represent autolysosomes and autophagosomes, respectively. The counts of both types of fluorescent dots increased significantly after CS treatment, while Tre treatment decreased the number of yellow fluorescent dots. These data suggest that Tre alleviates the CS-induced accumulation of autophagic substrates. 

Previous studies showed that Tre could augment lysosomal biogenesis and autophagic flux to ameliorate various disease phenotypes [23,36,39,40,41]. These effects were accompanied by the activation and nuclear translocation of TFEB. In this study, TFEB knockdown in MH-S cells reversed the protective effects of Tre against CS-induced autophagic flux damage. Tre upregulated Atg5 and Beclin 1 but downregulated LC3II in CS-treated MH-S cells, while TFEB knockdown downregulated the levels of all three proteins and inhibited autophagy (Figure 6E,F). TFEB knockdown also abolished the ability of Tre to abrogate the effects of CS on LAMP1 and CTSB levels and autophagic flux (Figure 6G–I and Figure S6B). Although the CS + Tre group further increased the colocalization of LAMP1 and LC3 as compared with the CS group, TFEB knockdown blocked this effect (Figure 6J). Moreover, TFEB knockdown abolished the ability of Tre to attenuate the accumulation of p62 and ubiquitinated proteins caused by CS. (Figure 6K–M). Collectively, these data suggest that Tre relieves CS-induced lysosome damage and restores autophagic substrate degradation through the activation of TFEB.

### 3.7. Tre Relieves CS-Induced Apoptosis and Inflammation through TFEB Activation

**Figure 7:**
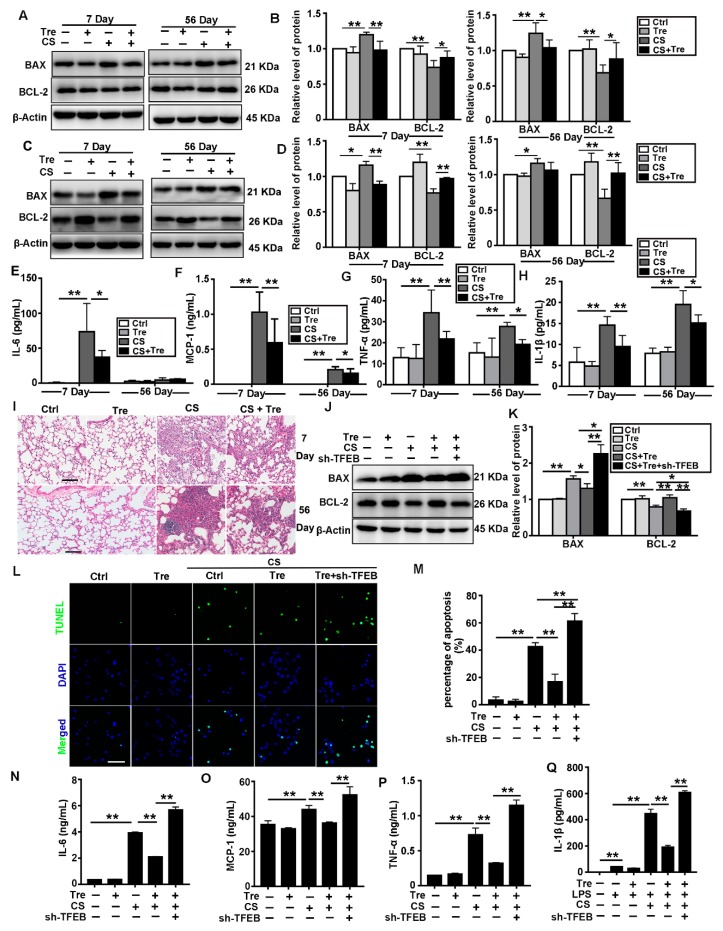
Tre may relieve CS-induced apoptosis and inflammation may through TFEB activation. (**A**,**B**) Immunoblotting analysis of BAX and BCL-2 protein levels in lungs on day 7 or 56 post CS instillation (*n* = 5). (**C**,**D**) Immunoblotting analysis of BAX and BCL-2 protein levels in AMs on day 7 or 56 post CS instillation (*n* = 3). (**E–H**) ELISA analysis of IL-6, MCP-1, TNF-α, and IL-1β levels in BALF on day 7 or 56 post CS instillation (*n* = 5 to 6). (**I**) H&E staining of mouse lungs on day 7 or 56 post-CS instillation (scale bar = 100 μm and *n* = 5). (**J**,**K**) Immunoblotting analysis of BAX and BCL-2 protein levels in MH-S cell lines (*n* = 3). (**L**,**M**) TUNEL (green) and DAPI (blue) staining and ratios of TUNEL-positive apoptotic MH-S cells (scale bar = 50 μm and *n* = 3). (**N**–**Q**) ELISA analysis of IL-6, MCP-1, TNF-α, and IL-1β levels in MH-S cell supernatant (*n* = 3). Data are presented as the mean ± SD. *, *p* < 0.05 and **, *p* < 0.01.

Autophagy is associated with apoptosis and inflammatory diseases. Moreover, a reduction in the apoptosis and the inflammatory responses can delay the progression of pulmonary fibrosis [42,43,44]. We found that CS treatment downregulated BCL-2 but upregulated BAX both in the lungs and the AMs of CS-treated mice. Tre markedly reduced the CS-induced effects (Figure 7A–D). Tre also alleviated the CS-induced increase in the Cas3 levels in the BALF (Figure S6C). The results of Elisa (IL-6, MCP-1, TNF-α, and IL-1β) in the BALF and HE in the lungs of mice showed that Tre alleviated CS-induced pulmonary inflammation (Figure 7E–I). Moreover, Tre markedly reduced the amount of apoptosis induced by CS treatment in MH-S cells, however, TFEB knockdown abrogated this antiapoptotic effect (Figure 7J–M). TFEB knockdown also neutralized the effects of Tre on CS-induced Cas3 levels (Figure S6D) and inflammatory cytokines (Figure 7N–Q). These observations demonstrate that Tre could relieve CS-induced apoptosis and inflammation through TFEB activation. 

### 3.8. Tre Relieves Pulmonary Fibrosis in Silicosis Model Mice

**Figure 8:**
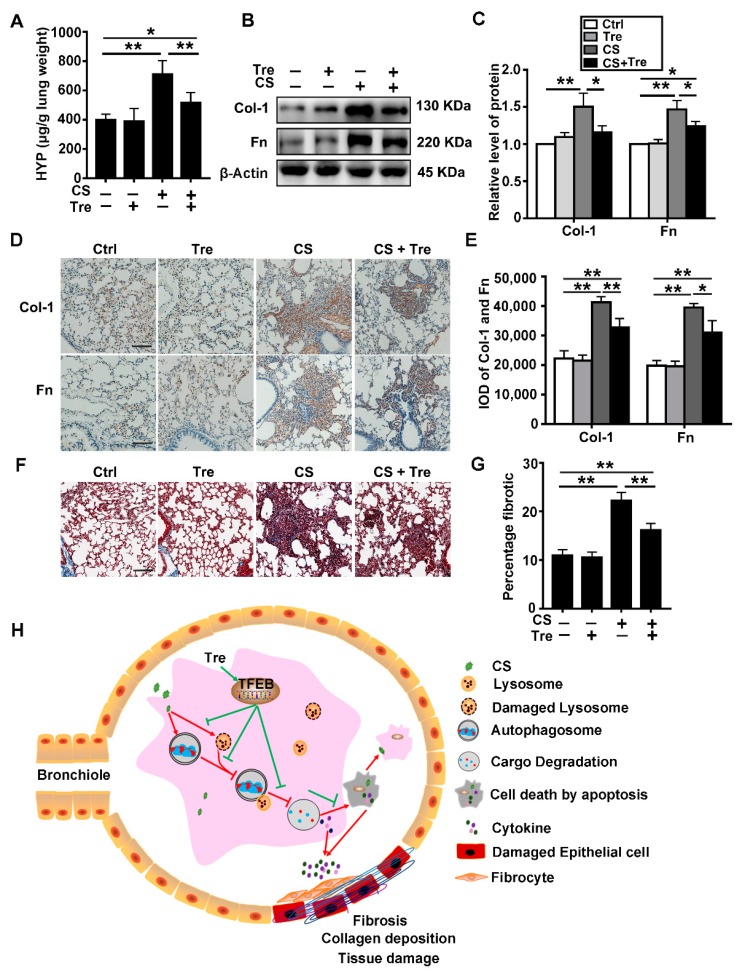
Tre relieves CS-induced pulmonary fibrosis. (**A**) HYP content in lung tissue was measured on day 56 post CS instillation (*n* = 5). (**B**,**C**) Immunoblotting analysis of Col-1 and Fn protein levels in mouse lungs on day 56 post CS treatment (*n* = 5 to 6). (**D**,**E**) Immunohistochemical staining and analysis of Col-1 and Fn levels in paraffin sections of mouse lung on day 56 post CS treatment. (**F**) Masson’s trichrome staining of mouse lungs on day 56 (scale bar = 100 μm and *n* = 5). (**G**) Fibrotic score analysis of the lung sections on day 56 post CS instillation. The fibrotic area is presented as a percentage. Data are presented as the mean ± SD. *, *p* < 0.05 and **, *p* < 0.01. (**H**) Illustration of the protective effects of Tre on CS-induced lung inflammation and fibrosis through the activation of the TFEB-dependent autophagy-lysosome machinery. CS impairs lysosomal function, inhibits autophagosome-lysosome fusion, and impairs autophagic substrate degradation. Tre induces TFEB nuclear translocation and improves the function of the autophagy-lysosomal system, leading to decreased apoptosis and secretion of inflammatory factors to alleviate CS-induced lung tissue injury and fibrosis.

Based on previous results, we explored the biological effects of Tre on CS-induced pulmonary fibrosis in a silicosis mouse model. We evaluated the effect of Tre on CS-induced lung fibrosis by analyzing the degree of collagen deposition in the lungs of mice by measuring the hydroxyproline (HYP) content (Figure 8A) and evaluating Masson’s trichrome stained lung sections (Figure 8F,G). We also measured the levels of Col-1 and Fn (fibrotic markers) in the lungs (Figure 8B–E). On the basis of these analyses, Tre reduced the levels of collagen deposition and fibrotic markers in the lungs of CS-treated mice at day 56 post CS treatment. Moreover, the body weight of CS-treated mice was regained faster by treatment with Tre as compared with the mice treated with CS alone (Figure S7). Taken together, these data show that Tre could alleviate pulmonary fibrosis in the lungs of CS-treated mice. 

## 4. Discussion

Silicosis is a progressive and chronic inflammatory lung disease. A reduction in apoptosis and inflammation could effectively delay the progression of fibrogenesis [44]. In this study, we demonstrated that CS induced TFEB nuclear localization and upregulated TFEB expression, which could be related to macrophage lysosomal disruption. TFEB overexpression or treatment with the TFEB activator Tre restored lysosomal function, accelerated autophagic substrate degradation, and enhanced autophagic flux in CS-treated AMs in vitro and in vivo. These treatments reduced CS-induced macrophage apoptosis, pulmonary inflammation, and fibrosis. On the basis of these results, we proposed a potential molecular mechanism for the protective effect of Tre against CS-induced pulmonary fibrosis through the regulation of the autophagy-lysosome system in AMs (Figure 8).

TFEB, a master regulator of the autophagy-lysosomal pathway, is closely associated with inflammation and fibrosis [17,45]. In resting cells, it is sequestered in the cytoplasm. Upon activation, TFEB is translocated into the nucleus, where it controls the expression of genes that regulate autophagosome formation and cargo degradation [46,47,48]. We found that TFEB is upregulated and translocated to the nucleus in the lungs of CS-treated mice, especially in the AMs (Figure 1). We studied the role of TFEB in CS-treated macrophages by knocking it down or overexpressing it in MH-S cells. Surprisingly, TFEB knockdown exacerbated CS-induced cell apoptosis and the inflammatory response (Figure 2D–K). Reports have indicated that AMs engulf particles through scavenger receptors, leading to lysosomal disruption and further TFEB nuclear translocation [4,31,49,50]. The nuclear translocation of TFEB in the AMs of mice with silicosis could serve a protective role against CS-induced lysosomal disruption. Conversely, knockout of TFEB dysregulates the innate immune response in LPS-injured macrophages [51]. Consistent with our hypothesis, studies in atherosclerosis and liver fibrosis have indicated that increased TFEB expression abrogates macrophage apoptosis and decreases proinflammatory cytokine levels, which reduces atherosclerosis plaque formation or fibrogenesis [20,24,52]. In this study, TFEB activation appeared to effectively alleviate CS-induced damage to AMs.

Tre, a disaccharide of glucose, is synthesized by some bacteria, yeast, fungi, certain plants, and invertebrate animals [53]. It is listed as a pharmaceutical reagent by the United States Pharmacopeia—National Formulary and Japanese Pharmacopeia, paving the way for its use in therapeutic products [54]. Recent studies showed that Tre could induce TFEB activation and reduce disease burden in a model of neurodegenerative disease [55]. We found Tre activated TFEB and restored the function of the autophagy-lysosomal system, in vivo and vitro. These data are consistent with the notion that Tre could induce lysophagy via the TFEB pathway [40]. TFEB knockdown counteracted the degradation of autophagic substrates induced by Tre in CS-treated animals (Figure 4, Figure 5 and Figure 6). Indeed, TFEB overexpression and Tre treatment improved lysosomal function in CS-treated macrophages. However, the exact mechanism and the role of lysophagy need to be further explored.

Autophagy, a “self-eating” catabolic process, is markedly essential for maintaining cell homeostasis and normal cellular function [56,57]. Our previous study showed activated autophagy and increased autophagosomes in AMs of silicosis patients and mouse model [13,15]. Studies reported that inhibition of autophagy in macrophages could relieve CS-induced pulmonary fibrosis [58,59]. However, Atg5 knockdown in mice showed that impairment of autophagy aggravated CS-induced pulmonary inflammation and fibrosis [14,15]. The results from genetically modified mice showed that autophagy is vital for maintaining lung homeostasis. Increasing LC3II expression together with enhanced autophagic flux alleviates CS-induced pulmonary fibrosis by promoting autophagy in AMs [15]. However, in this study, we found that LC3II expression was decreased in the CS + Tre-treated AMs as compared with the CS-treated alone. We reasoned that Tre might restore lysosomal function that was disrupted by CS particles, enhance the autophagic flux, and promote the degradation of autophagic substrates [60]. Indeed, the results with mRFP-GFP-LC3 adenovirus-transfected MH-S cells verified that Tre could enhance autophagic flux and alleviate CS-induced autophagosome accumulation. Previously, our group demonstrated that promoting mitophagy in AMs could alleviate CS-induced tissue injury [15]. In this study, we found that Tre could protect lysosomes to alleviate the detrimental effects induced by CS. All these conclusions are based on the premise that autophagic flux was not completely blocked in our experimental systems. A regular autophagic flux is necessary for maintaining homeostasis in cells [61].

Lysosomes are acid organelles for degradation at the end of the autophagic flux. Lysosomal defects disturb the degradation of autophagic substrates, leading to the accumulation of autophagosomes [62,63]. In the AMs from silicosis patients, lysosomal defects can lead to the inhibition of autophagic degradation [13]. Engulfed CS in these AMs can destroy the lysosomes, thereby blocking autophagic flux. This blockage could lead to the accumulation of autophagosomes, macrophage apoptosis, and an increased inflammatory response. In this study, we used BAF and CQ to measure autophagosomes synthesis and degradation through changes in LC3-II levels. We found that the elevated numbers of autophagosomes in CS-treated MH-S cells resulted from both the activation of autophagy and inhibition of cargo degradation (Figure S5) [64]. Both TFEB overexpression and Tre treatment could improve lysosomal function and accelerate autophagosome degradation. These effects alleviated the detrimental effects induced by CS. Silicosis is closely associated with apoptosis and the release of proinflammatory and profibrotic cytokines by AMs. A reduction in apoptosis and inflammation could effectively delay the progression of pulmonary fibrosis [44]. The secretion of cytokines, including IL-1β, IL-6, TNF-α, and MCP-1, plays a certain role in the pathogenesis of pulmonary fibrosis [29]. Indeed, silica-induced collagen deposition is almost completely prevented by treatment with an anti-TNF antibody [65]. Treatment with Tre effectively abrogates Cl_2_- and LPS-induced pulmonary inflammatory cascades, in the lung [66,67]. In this study, we found that TFEB overexpression or Tre treatment reduced CS-induced apoptosis and inflammation. In contrast, TFEB knockdown reversed these mitigative effects. Tre also effectively alleviated pulmonary fibrosis in the CS-treated mice. On the basis of these results, we reason that Tre exerts anti-inflammatory and antifibrotic effects by restoring lysosomal function and promoting autophagic substrate degradation. 

In summary, the findings of this study suggest that autophagy-lysosomal system dysfunction could represent a therapeutic target against CS-induced damage. Moreover, we demonstrated a potential therapeutic strategy (i.e., Tre) that could improve lysosomal function and accelerate autophagosome degradation. This strategy could reduce the apoptosis in AMs and alleviate the inflammatory responses and fibrogenesis through the activation of TFEB-mediated autophagy-lysosome machinery. We showed that both TFEB overexpression and Tre treatment improved lysosomal function in CS-treated MH-S cells. However, understanding the exact mechanism requires further investigation. Selective autophagy of organelles (e.g., mitophagy, lysophagy, and reticulophagy) is critical for the regulation of cellular homeostasis in organisms from yeast to humans [68]. Future experiments should focus on the role of selective autophagy and the relationship between mitophagy, lysophagy, and reticulophagy in Tre-treated silicosis or TFEB-overexpressing or silenced mouse models.

## Supplementary Materials

The following are available online at https://www.mdpi.com/2073-4409/9/1/122/s1, Figure S1: Administration method of trehalose in experimental mouse model of silicosis; Figure S2: Transfection efficiency of TFEB knockdown and overexpression lentivirus; Figure S3: Viability of MH-S incubated with different concentrations of BAF, CQ, and 3MA; Figure S4: Effects of CS and/or Tre on cell viabilities in MH-S cells after 12 h treatment; Figure S5: Immunoblotting analysis of LC3II after CS treatment with or without Bafilomycin A1/CQ; Figure S6: Immunoblotting analysis of protein CTSB and Cas3 in BALF or in cell supernatant; Figure S7: The body weights of animals after CS and Tre administration.
