# Supplementary material for: Trehalose Alleviates Crystalline Silica-Induced Pulmonary Fibrosis via Activation of the TFEB-Mediated Autophagy-Lysosomal System in Alveolar Macrophages
Source: Cells. 2020 Jan 4;9(1):122. doi: 10.3390/cells9010122 (PMC7016807; doi:10.3390/cells9010122)
Supplement: Supplementary file 1 [file cells-09-00122-s001.pdf]

Supplementary Materials:

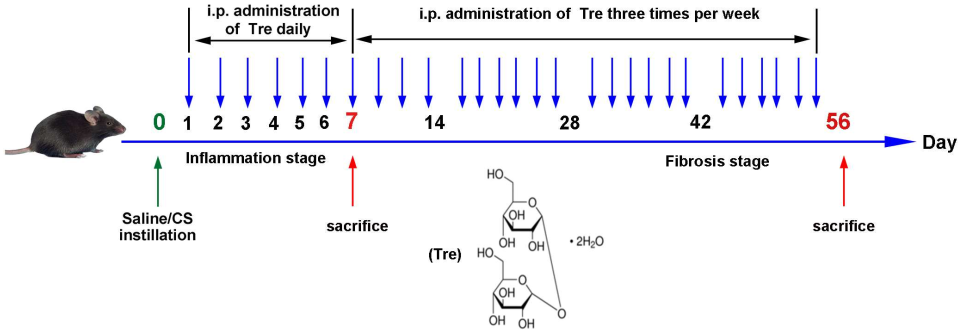

Figure S1 Administration method of Trehalose in experimental mouse model of silicosis. Trehalose (2 g/kg body weight) or saline was given to C57BL/6 mice by daily intraperitoneally (i.p.) administration to C57BL/6 mice after saline or CS injection for 7 days model mice. For 56 days model mice, after 7 days i.p. administration, mice were administered 3 times per week (n=14 per group).

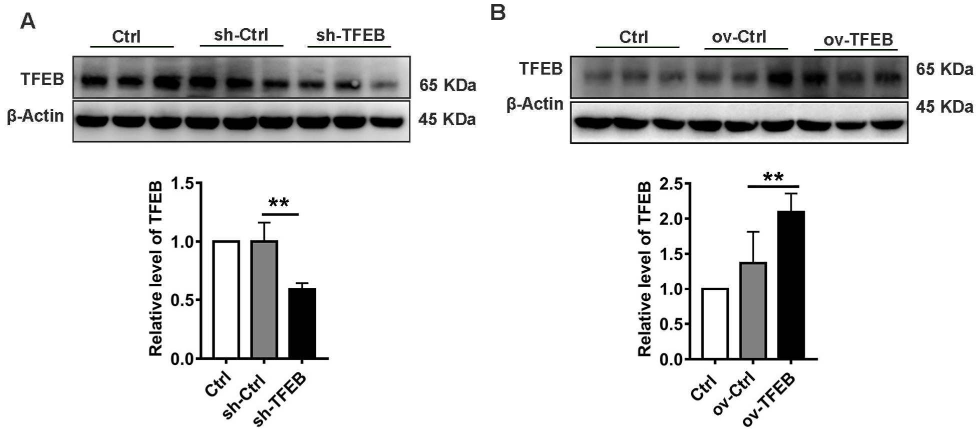

Figure S2 Transfection efficiency of TFEB knockdown and overexpression lentivirus (A) Immunoblotting analysis of TFEB knockdown transfection efficiency **(B)** Immunoblotting analysis of TFEB overexpression transfection efficiency (n=3). **, $P < 0.01$. Data are shown as mean ± SD.

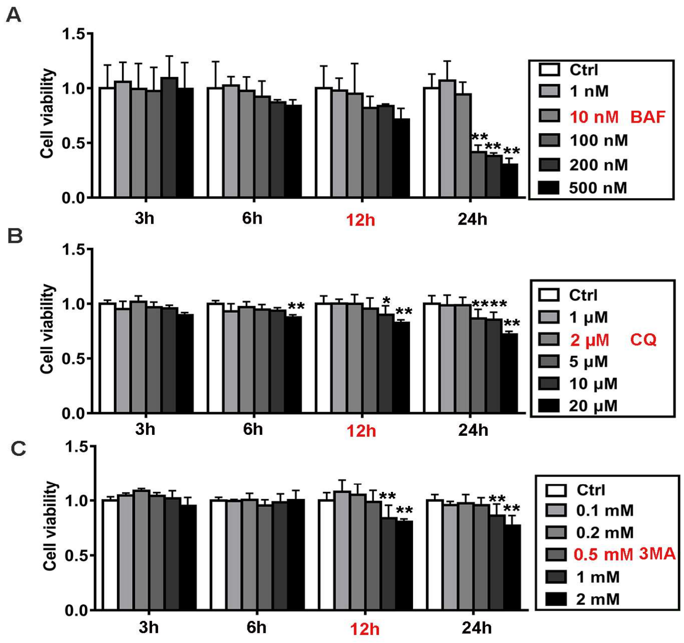

Figure S3 Viability of MH-S incubated with different concentrations of BAF **(A)**, CQ **(B)**, 3MA **(C)** was detected by the CCK-8 assay (n=4-6). Compared with Ctrl. *, $P < 0.05$; **, $P < 0.01$. Data are shown as mean ± SD.

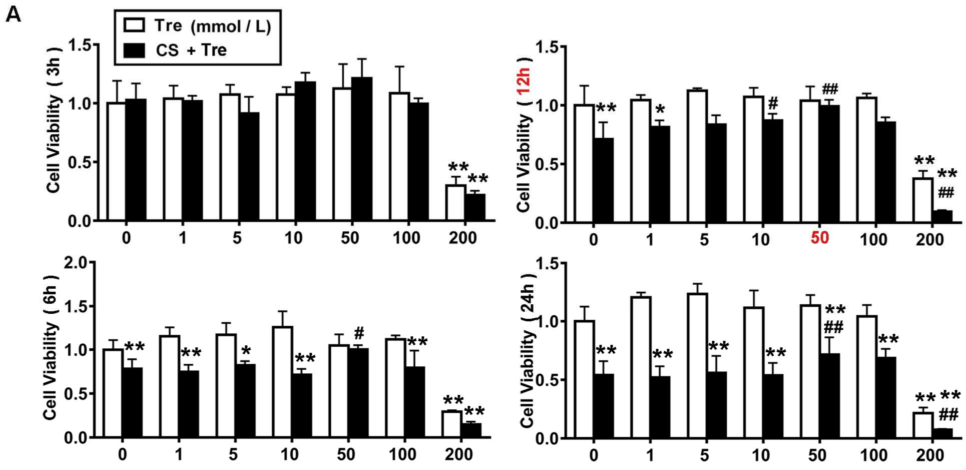

Figure S4 Effects of CS and/or Tre on cell viabilities in MH-S cells after 12 h treatment. Cells were incubated with a range of Tre concentrations (0, 1, 5, 10, 50, 100 and 200 mmol/L) and/or 50 μg/cm2 CS for 12 h to determine the cell survival. White, different concentrations of trehalose. Black, cells were treated with 50 μg/cm2 CS and different concentrations of trehalose (n=4). Compared with Tre (0 mmol / L) *, $P < 0.05$; **, $P < 0.01$; compared with CS (50 μg/cm2) + Tre (0 mmol/L). #, $P < 0.05$; ##, $P < 0.01$. Data are shown as mean ± SD.

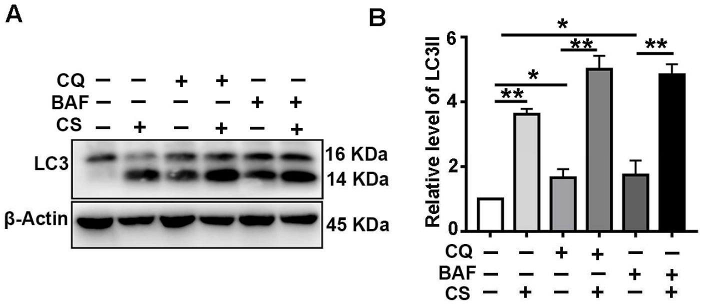

Figure S5 A&B MH-S cells were exposed to CS (50 μg/cm2) with or without Bafilomycin A1 (10 nmol/L) or CQ (2 μmol/L), immunoblotting analysis of LC3II (n=4). *, $P < 0.05$; **, $P < 0.01$. Data are shown as mean ± SD.

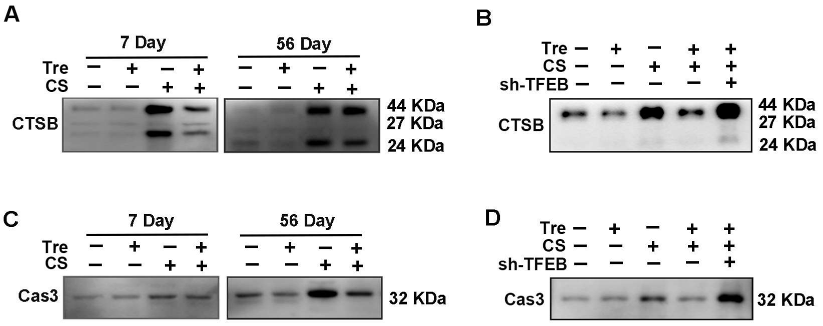

Figure S6 (A) Immunoblotting analysis of protein CTSB in BALF at day 7 and 56 after CS and Tre administration (n=4-5). **(B)** Immunoblotting analysis of protein CTSB in cell supernatant after CS and Tre treatment 12h (n=3). **(C)** Immunoblotting analysis of protein Cas3 in BALF at day 7 and 56 after CS and Tre administration (n=4-5). **(D)** Immunoblotting analysis of protein Cas3 in cell supernatant after CS and Tre treatment 12h (n=3).

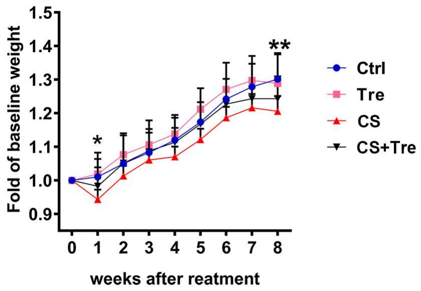

Figure S7 Animals body weights after CS and Tre administration (n=10). Compared with Ctrl group. *, $P < 0.05$; **, $P < 0.01$. Data are shown as mean ± SD.
